# Trauma, personality structure and psychopathology: a cartography of psychodynamic constructs

**DOI:** 10.1186/s40479-025-00308-0

**Published:** 2025-08-18

**Authors:** Jürgen Fuchshuber, Victor Blüml, Nestor Kapusta, Henriette Löffler-Stastka, Johanna Alexopoulos, Elisa Renner, Hugo Senra, Human-Friedrich Unterrainer

**Affiliations:** 1 Department of Psychoanalysis and Psychotherapy, Medical University Vienna, Vienna, Austria; 2 Comprehensive Center for Clinical Neurosciences and Mental Health, Medical University Vienna, Vienna, Austria; 3 Center for Integrative Addiction Research (CIAR), Grüner Kreis Society, Vienna, Austria; 4 Institute of Psychology, Sigmund Freud University, Freudplatz 1, 1020 Vienna, Austria; 5 IEETA, University of Aveiro, Aveiro, Portugal; 6 School of Health and Social Care, University of Essex, Essex, UK; 7 Institute of Psychology, University of Graz, Graz, Austria; 8 University Clinic for Psychiatry and Psychotherapeutic Medicine, Medical University Graz, Graz, Austria; 9 Department of Religious Studies, University of Vienna, Vienna, Austria; 10 Faculty of Psychotherapy Science, Sigmund Freud University, Vienna, Austria

**Keywords:** Network analysis, Primary affects, Psychopathological symptoms, Attachment security, Personality

## Abstract

**Background:**

In this study, network analysis technique is applied to dissect the links between personality organization, reflective functioning, attachment security, primary affective traits, childhood trauma and psychopathological symptoms.

**Methods:**

A total sample of 498 (77% female) participants from the general population was investigated. A cross-sectional network between personality organization [IPO-16], hypomentalizing [RFQ-6], attachment [ECR-RD8]), primary affective traits [BANPS-GL], depression, anxiety and somatization symptoms [BSI-18], addiction [WHO-ASSIST] and childhood trauma [CTQ] was estimated via the EBICglasso and relimp algorithm. Regularized partial correlation edge weights, node centrality, predictability, bridge centrality, relative importance and stability coefficients were analyzed.

**Results:**

We observed personality organization, SADNESS and hypomentalizing as the most influential personality constructs within the investigated network. Personality organization and hypomentalizing were also observed as nodes with the highest bridge centrality, signifying their potential relevance as mediator between trauma, affect and psychiatric symptom severity.

**Conclusions:**

The results enable a data-driven, in-depth examination of the complex and often reciprocal relations among psychopathological symptoms, childhood adversity and psychodynamic personality constructs. Our observations highlight critical interconnections among childhood trauma, primary affects, personality functioning and psychopathology and pinpoint personality organization, hypomentalizing and SADNESS as central psychodynamic personality constructs.

**Supplementary Information:**

The online version contains supplementary material available at 10.1186/s40479-025-00308-0.

## Background

### Network analysis and psychodynamic research

In the last decade, many scientific disciplines saw a surge regarding the introduction of network scientific approaches into their methodology. The increase of publications relying on network analytical tools specifically in the field clinical psychology [1, 2], implies a fertile ground for this statistical approach and its possibilities to think about psychopathology in terms of complex systems.

Psychoanalysis has seen repeated attempts to theoretically integrate principles of cybernetics [3, 4] or network and system theory [5–17]. The existing wealth of conceptual works is in stark contrast to the relatively rare empirical applications of network analytic techniques in psychodynamic research. Nevertheless, the existing research using these set of methods demonstrated the versatile possibilities of the network analytic approach: Recent examples include investigations on pathways connecting hypomentalizing (HM) impairments and conduct problems [18], associations between psychodynamic conflicts and levels of structural integration [19], the relationship between somatization, depression, conflicts and personality structure [20] and similarities and differences of different psychometric measures for personality structure [21]. What these studies have in common is that they enabled a data driven glance into the complex systems of psychodynamic concepts, while taking into account their interconnectivity. In their most recent study, Vierl and colleagues [22] have investigated the interplay between psychopathological symptoms, interpersonal problems, modes of conflict and personality functioning. Their results indicated the personality organization (PO) subdomain identity diffusion as the most influential node within the psychodynamic constructs, while self-perception and passive self-worth conflict showed the strongest relation to psychopathological symptom severity.

### Personality functioning

Both the DSM-5 and upcoming ICD-11 include a new diagnostic emphasis on personality functioning. The DSM-5's alternative model of personality disorders (AMPD), proposes personality functioning as central diagnostic criteria for personality disorders. In the AMPD, personality functioning is split into intrapersonal (identity, self-direction) and interpersonal (empathy, intimacy) aspects [23]. Similarly, the ICD-11 defines personality disorders by problems in functioning of aspects of the self (e.g., identity, self-worth, accuracy of self-view, self-direction) and/or interpersonal dysfunction (e.g., ability to maintain satisfying relationships, understand others' perspectives, manage conflict) [24]. This perspective converges considerably with psychoanalytic research regarding the clinical significance of personality structure [25], which hypothesizes that psychiatric symptoms can occur as a result of an impairment of personality structure. These impairments are assumed to be associated with disturbances (e.g. trauma, deprivation or innate vulnerabilities) during the development of the individual. In this context, personality structure is defined as relative time stable set of ego functions that ensure the maintenance of inner equilibrium and relationships to others [26].

Kernberg's work significantly advanced psychoanalytic theory on personality structure. Influenced by Melanie Klein, Edith Jacobson and W.R.D. Fairbairn, he integrated drive theory, ego psychology and object relations theory into a cohesive framework. Kernberg [27–29] argues that internalized object relations, consisting of self-representation, object representation and affect, form the basic components of drives and psychological structures (id, ego, superego). These relations are influenced by early experiences but also by the interaction between the infant’s temperament and its environment. The internalization process of object relations evolves from the infant's undifferentiated ego-id to a mature tripartite structure [30, 31] and takes place in consecutive layering sequences, mediated by the affective memory storage linked to hippocampal networks [27]. This process involves introjection, identification and ego-identity, finally leading to the precipitation of relative stable psychic structures. Kernberg’s [32] model of personality organization (PO) identifies three major dimensions of functioning: (1) Coherence of identity, (2) Maturity of defense mechanisms and (3) Ability to test reality. Severe identity diffusion, primitive defenses and intact reality testing characterize “borderline organization.” Additional impairments in reality testing define psychotic personality organization, while neurotic personality organization shows minimal deficits in all areas. These concepts are interconnected, representing a continuum of personality functioning [33].

To add to that, Kernberg’s more recent formalizations of PO note further functional differentiations, including the quality of object relations, coping, mature defenses and rigidity, aggression and moral values [34, 35], enabling a more fine grained personality assessment.

### Attachment

In its conception historically intertwined with psychoanalysis, the field of attachment theory suggests that affect regulation development relies on early nonverbal communication between infants and caregivers [35–37]. Ideally, caregivers perceive and co-regulate infants' nonverbal affective expressions through mirroring and comfort, aiding infants in managing intense emotions. This process helps infants develop positive internal models of self and others, supporting autonomous emotional regulation and exploration [38]. Secure attachment fosters stable relationships and emotional regulation with others. Conversely, traumatic early experiences lead to negative internal models and insecure attachments, hindering emotional regulation and relationship formation [39].

Ainsworth and Bell [40] classified insecure attachments in infants as anxious-ambivalent, anxious-avoidant and disorganized, corresponding to Bartholomew and Horowitz’s [41] adult classifications: anxious-preoccupied, dismissive-avoidant and fearful-avoidant. Longitudinal studies show early attachment styles are stable into adulthood [42, 43]. Regarding psychometric (self-report) research this categorization was further boiled down via factor analytic investigations [44], resulting in the differentiation between: attachment anxiety—an overactive pattern—which is defined by the fear of interpersonal rejection and the excessive search for closeness and recognition by others, combined with low self-confidence; and, attachment avoidance—a deactivation pattern—which is associated with fears of closeness and interpersonal dependence [45].

### Mentalization

Mentalization or reflective functioning describes the cognitive process by which people, both consciously and unconsciously, interpret their own actions and those of others as meaningful based on intentional mental states like desires, needs, emotions, beliefs and reasons [46]. Conceptually based within the Theory of Mind, mentalization emphasizes more strongly on the emotional and relational comprehension of oneself and others [47]. This approach highlights skills that help individuals not only navigate social interactions effectively but also build a richer, more stable sense of identity. Significant correlations between the ability to infer mental states and a range of psychopathological disorders have been reported in meta-analyses [48, 49]. Mentalization can be assessed using a range of methods, including semi-structured interviews, performance-based tasks, and self-report questionnaires. Fonagy and colleagues introduced the Reflective Functioning Questionnaire (RFQ; 50), a brief self-report tool to evaluate an individual's ability to interpret mental states of themselves and others, known as reflective functioning or mentalization. In its most recent iteration [51], the RFQ-6 measures individual differences in hypomentalizing (HM). Linked to the mentalization-based approach to psychological development and psychopathology, Fonagy and colleagues [52] have highlighted the theory of epistemic trust, which is defined as the capability to accept social/communicated knowledge perceived as trustworthy and meaningful. This approach conceptualizes features of personality disorders as being result of an “emergency mode of social understanding” that the individual develops as an adaptive consequence to their environment. The authors also spotlight recent observational studies suggesting dysfunctional epistemic strategies as factors in mental health vulnerability [53].

### Primary affects

Iterating Freudian drive theory, contemporary (neuro-) psychoanalytic findings emphasize biologically hard-wired motivational systems, bordering between physiological and psychological experience. These circuits are organized into pleasurable and unpleasurable feeling systems, reflecting the basic distinction of entropy and negative-entropy regarding homeostatic needs of the organism. Panksepp’s primary affect model [54–56] further distinguishes seven emotion networks, which arise from midbrain structures like the periaqueductal gray and expand into the limbic forebrain. Four of those systems have evolutionary reptilian roots. These comprise SEEKING, which mediates appetitive foraging; FEAR, mediating freeze and flight behavior; LUST (sexual excitement, pleasure and desire) and ANGER (aggressive attack behaviors). For mammalian brains, Panksepp’s theory proposes additionally networks for SADNESS (mourning, grief and panic), CARE (nurturing) and PLAY (playful behavior and social joy). For a detailed description see Panksepp [54] and Solms [57].

### General framework

Aiming to build an integrative framework, neuropsychoanalysis posits a dynamic, multi-layered structure comprising primary, secondary and tertiary processes [58]. These layers correspond to different levels of consciousness: anoetic (non-language based forms of experience), noetic (knowledge and learning based forms of experience) and autonoetic (referring to the ability of self-awareness and to reflect the existence of oneself as a temporal entity) [59]. This model aligns with MacLean’s “triune brain” theory, which includes the reptilian, paleomammalian (limbic system) and neomammalian (neocortex) layers [60].

Primary processes are subcortical and anoetic. They include basic drives (hunger, thirst), sensory affects (pleasure, disgust) and primary emotions (SEEKING, ANGER, LUST, FEAR, PANIC/GRIEF, CARE and PLAY). These are seen as the foundation of the behavioral motivation system. Secondary processes involve noetic operations, largely unconscious behavioral traits, like personality functions, object relations and attachment patterns, linked to cognition, memory and procedural memory, primarily rooted in the basal ganglia and limbic structures. Tertiary processes, based within the neocortex, involve autonoetic operations, including abstract thinking, mentalization and mindfulness.

The neuropsychoanalytic model implies continuous feedback loops expressed between these layers. As this model proposes a complex and dynamic system, they might be interpreted in terms of the epistemologically linked fields of chaos and complexity theory [16, 61]. This suggests mammalian brains self-organize but can also experience perturbations, leading e.g. to psychiatric disorders. Early relationship experiences significantly influence the development of these processes, acting as *tuning variables* for the brain’s adaptive responses to environmental changes.

### Childhood trauma

Interpersonal childhood trauma has been a subject of scientific investigation for well over a century [62, 63]. Experiences of interpersonal childhood trauma are defined as exposure to sexual, physical and emotional abuse and neglect prior to the age of 18 [64] and its role as a significant transdiagnostic risk factor for various psychiatric disorders is well-documented [65–68]. In a psychoanalytic context, trauma is understood as an event so intense and overwhelming that it is impossible for the subject to integrate this experience within the symbolic structure [69]. Further research point towards the eroding effects of traumatic childhood experiences regarding cognitive and affective functioning, which might mediate its relationship with psychiatric symptoms [70–73]. Resonating this line of evidence, previous psychometric research employing path analysis indicated that memories of traumatic childhood experiences lead to less adult attachment security and more severe deficits in PO, which in turn lead to increased negative and decreased positive primary affective traits, as well as more severe psychopathological symptoms in terms of addictive behavior, depression, anxiety and somatization [74, 75].

### Study aims

In the present study we aim to expand upon findings regarding the ties between the concepts outlined above by employing a regularized partial correlation network analysis on data from an extensive sample of the general population. The aim for this procedure is to dissect the associations within this complex conceptual system. Specifically, we try to investigate the following umbrella hypotheses: (1) Childhood trauma is associated with impairments in personality structure (PO, RF, attachment security), primary affects dispositions and psychopathology. (2) Personality structure and primary affect dispositions are interrelated. (3) Personality structure and primary affect dispositions are associated with psychopathological symptoms.

Based upon the resulting network structure, this study will then be able to estimate the most influential nodes within the network as well as identify bridges between them. Furthermore, we will be able to describe meaningful relationship patterns among the variables. In a final step, we will use the relative importance technique to study the relationships between the concepts regarding potential reciprocal relationships.

Overall, the study aims to be a proof-of-concept for the network analytical study of psychodynamic concepts.

## Material and methods

### Sample and procedure

The final sample comprised 498 German-speaking young adults (*sex* = 76.5% female; *age range*: 18–85 years, *M* = 27.89, *SD* = 11.52). To participate, individuals had to be over 18 years old, fluent in German and have completed all the questionnaires. Initially, there were 966 participants, but 464 were excluded due to incomplete surveys, and 4 were excluded for providing either an implausible age information (age = 102 years; n = 1) or the age information indicated an age < 18 (n = 3). Recruitment was conducted through public announcements at the University of Graz, a student email distribution list and social networks such as Instagram and Facebook. The questionnaires were administered via the online survey platform LimeSurvey. Informed consent was obtained from all participants before they began the survey, which included various sociodemographic questions (e.g., age, gender, education, psychiatric diagnosis) and standardized test procedures. Participants were not compensated and remained anonymous throughout the study. The research adhered to the Declaration of Helsinki and received ethical approval from the Ethics Committee of the University of Graz, Austria. Recruitment took place from September 2023 to March 2024. LimeSurvey settings were configured to prevent missing data.

### Psychometric assessment

#### Sociodemographic information

After providing informed consent, participants completed a sociodemographic questionnaire collecting all personal data relevant to the study. This questionnaire included questions on age, gender, marital status, education level and field of study, current occupation or training, presence of psychiatric disorders, medication, as well as country of origin and language skills.

#### Childhood trauma

The German version of the *Childhood Trauma Questionnaire* (CTQ; 76) translated by Wingenfeld, Spitzer [77] is a self-report measure consisting of 28 items that assess traumatizing experiences during childhood. The questionnaire includes a total childhood trauma scale as well as the subscales: emotional Abuse, physical Abuse, sexual abuse, physical neglect, as well as emotional neglect. Respondents rate their recalled experiences on a Likert scale ranging from 1 (“never”) to 5 (“very often”), with higher scores indicating more severe instances of abuse or neglect. The internal consistencies of the total scale was found to be excellent, with Cronbach’s *α* ≥ 0.89 [77].

#### Primary affects

The German Version of the *Brief Affective Neuroscience Personality* Scales including a LUST Scale (BANPS-GL; 78) is a self-report questionnaire which covers all seven primary affects identified by Jaak Panksepp [79], including the subscales PLAY, CARE, SEEKING, ANGER, FEAR and SADNESS as well as an additional scale for the dimension LUST. The original Brief Affective Neuroscience Personality Scales (BANPS; 80) represents a shortened version of the Affective Neuroscience Personality Scales [81]. The BANPS-GL consists of 38 items and is rated on a 5-point scale from (1) strongly disagree to (5) strongly agree. The instrument previously provided acceptable to good internal consistencies ranging from Cronbach’s *α* = 0.69 (CARE) to *α* = 0.85 (SADNESS) [78].

#### Attachment

The abbreviated version of the *Experiences in Close Relationships-Revised* (ECR-RD8; 82) is an established self-report tool for the assessment of attachment insecurity in relation to anxiety (AX) and avoidance (AV) attachment behavior. The questionnaire contains eight items rated on a Likert scale from “strongly disagree” (1) to “strongly agree” (7). The short version achieves good to excellent reliability in its German version. The internal consistency, calculated using *McDonald’s ω* was 0.87 for anxiety and 0.91 for avoidance [82].

#### Personality organization

The *16-Item Inventory of Personality Organization* (IPO-16; German version by 33) is a self-report measurement of deficits within personality structure. The questionnaire is theoretically grounded in Otto Kernberg ‘s [29] model of personality organization. The IPO-16 is comprised of three subscales: (1) “Identity Diffusion,” which measures the integrity of the representations of oneself and others; (2) Dominance of primitive defense mechanisms such as splitting, denial, projection and dissociation (“Primitive Defense”); (3) the capacity to differentiate between internal and external stimuli (“Reality Testing”). A total score of Structural Deficits can be generated. The items are rated on a 5-point Likert scale ranging from 1 (“never”) to 5 (“always”). Internal consistencies for the total scale was previously estimated as excellent (*Cronbach’s α* = 0.85; 33).

#### Reflective functioning

The *Reflective Functioning Questionnaire* (RFQ; 51) was applied in its 6-item short version. The self-report questionnaire aims to assess hypomentalizing with higher values indicating stronger tendencies towards hypomentalizing and is rated on a 7-point Likert scale (“do not agree at all” = 1 to “agree completely” = 7). The 6-item version has shown good internal consistencies with *McDonald’s ω* ranging from 79 to 0.82 [51].

### Psychopathological symptoms

#### General symptom severity

The *Brief Symptom Inventory* (BSI-18; 83) consists of 18 items assessing the amount of symptom burden over the past 7 days. The German version was translated by Spitzer et al. [84]. The self-report measurement includes the subscales depression, anxiety and somatization. Items are rated on a five-point Likert scale ranging from 0 “absolutely not” to 4 “very strong.” A total score “Global Severity Index” can be generated by adding the scores of every item. Previously, all scales showed acceptable to good internal consistencies, with *Cronbach’s alpha* ranging from 0.64 (somatization) to 0.93 (depression) [84].

#### Substance use disorder

The *World Health Organization's Alcohol, Smoking and Substance Involvement Screening Test* (ASSIST; 85) is a standardized tool for identifying psychoactive substance use and related problems. For this online study, the ASSIST was adapted as a self-report questionnaire. It assesses lifetime use and abuse symptoms for 10 substance groups, including tobacco, alcohol, cannabis and others. Symptoms are rated on a 7-point Likert scale (0 = ”never” to 6 = ”daily or almost daily”) for frequency, craving, problems and disappointed expectations (questions 2–5). Questions 6–8 use a 3-point scale (0 = ”no, never” to 6 = ”yes, in the last 3 months”) for concerns, failed attempts to cut down and drug injection. In earlier research, the total score showed excellent internal consistency (Cronbach’s alpha = 0.94; 86).

#### Statistical analyses

We used SPSS 29.0 for data management, descriptive statistics and bivariate correlations, employing Pearson product-moment correlations for bivariate associations with two-tailed *p* values. Network analysis was conducted in RStudio 4.0.3. Our approach utilized the Extended Bayesian Information Criterion for graphical Lasso (EBICglasso) methodology [87, 88] with the corMethod = “cor_auto” [89] and a hyperparameter γ = 0.5. The network estimation was computed using the qgraph package in R [90]. Our network comprised 16 nodes. For visualization, we utilized an adapted Fruchterman-Reingold algorithm for node placement [91, 92]. No specific minimum, maximum or cut values have been used for network visualization.

To determine the key nodes within the network, we employed centrality indices calculated through the centrality function of the qgraph package. Besides the edge weights we investigated the following measures: (1) Expected Influence (EI) which is the aggregate weight of edges a node has with every other node in the network and also considers negative links of variables [93]. (2) Centrality strength which sums the absolute weights of all edges directly connected to the respective node [94]. (3) Predictability, which assesses the proportion of variance of a node explained by its adjacent nodes [95]. (4) We calculated Bridge Strength (BS) using the bridge function in the network-tools R package ([96]; version 1.2.3), defined by the sum of weights of edges linking a node to nodes in a different community to pinpoint nodes that serve as connection hubs between psychodynamic constructs and psychopathology [97]. The communities were defined by their respective theoretical umbrella concepts (attachment, hypomentalizing, childhood, negative and positive affect, as well as psychiatric symptoms).

To assess stability of the network and the precision of our estimated parameters, we employed bootstrapping techniques (with 2000 bootstrap samples via the bootnet package in R ([98]; version 1.4.3). To investigate the accuracy and robustness of the edge weights, we derived 95% confidence intervals (CIs) for each edge's estimated value.

For the analysis of the stability of investigated network measures (EI, CS and Bridge centrality), we utilized the correlation-stability coefficient (CS-coefficient), which measures how much of the dataset can be omitted while still ensuring a correlation of at least 0.7 with the original data, with 95% confidence. A CS-coefficient above 0.50 is desirable for interpreting a network as stable, whereas a value below 0.25 suggests that the network's centrality order is unstable [89]. The code and data matrix used in our analysis is available in the Supplements.

Finally, we used the relative importance (*relimp*) option in the *estimateNetwork* function of the bootnet package, to study the relationships between the concepts regarding potential reciprocal relationships. In a relative importance network, the edges signify the explained variance (*R*^*2*^) contributed by a single predictor after accounting for the relationship between the two variables, along with the direct influence one variable has on another, adjusted for all other variables [99]. For better clarity a cut-off for connections < 0.15 was employed in the visualization [100].

## Results

### Sample characteristics

The detailed descriptive characteristics of the sample are presented in Table 1. The data analysis included 498 individuals who completed the entire test battery completely and with consent. The sample was predominantly composed of German-speaking individuals from Germany, Austria and Switzerland, accounting for 91.2% of participants. Age ranged from 18 to 85 years, with an average age of 27.89 years (*SD* = 11.52). A majority of participants (76.5%) were female at birth. 91.8% had at least a high school education and 15.1% of participants reported having a diagnosed psychiatric condition.

**Table 1:** Sample descriptives

	M	Range	SD
Age	27.89	18–85	11.52

	Category	Frequency	%
Sex	Female	381	76.5
	Male	116	23.3
	Intersex	1	0.2
Gender	Female	366	73.5
	Male	115	23.1
	Diverse	17	3.4
Sexual orientation	Heterosexual	363	72.9
	Bisexual	73	14.7
	Homosexual	27	5.4
	Other	53	6
Educational status	No degree	1	0.2
	Compulsory education	4	0.8
	Technical school	29	5.8
	High school diploma	272	54.6
	Bachelor	109	21.9
	Master	68	13.7
	Doctorate	8	1.6
	Other	7	1.4
Occupation	School	2	0.4
	Apprenticeship	6	1.2
	University Studies	328	65.9
	Employee	110	22.1
	Self-employed	22	4.4
	Parental Leave	4	0.8
	Retirement	5	1
	Unemployed	14	2.8
	Part-time Job	7	1.4
Relationship status	Single	271	54.4
	Current relationship	165	33.1
	Married	52	10.4
	Divorced	10	2
Origin	Germanophone	454	91.2
	Other	44	8.8
Diagnosed with mental disorder	Yes	75	15.1

### Zero order correlations

As detailed in supplementary Table 1 and visualized in supplementary Fig. 1 we observed a dense pattern of interrelations between the investigated constructs. Childhood trauma showed a correlative pattern characterized by positive links to psychopathology and negative affect, attachment insecurity, as well as hypomentalizing (HM) and PO deficits (*r* =.21–0.50). With the exception of SEEKING, negative correlations were observed between childhood trauma and positive primary affects (*r* = −.15 to − 0.31).

In general, positive primary affects exhibited positive correlations amongst each other (*r* =.19–0.42) and negative correlations with negative affects (*r* = −.13 to − 0.36). While ANGER was dissociated from positive affect, FEAR was negatively linked to LUST (*r* = −.23) and CARE (r = −.16). In turn, negative emotions showed positive intercorrelations with each other (*r* =.27–.64). HM, PO and insecure attachment exhibited generally negative links with positive emotions (*r* = −.12 to − 0.48) and positive links with negative emotions (*r* =.15–0.44). Regarding its relationship with psychiatric symptoms, we generally observed the expected pattern of a negative relationships between positive affects and symptoms load (r = −.10 to − 0.38), as well as positive relationships between negative affects, HM, attachment and PO with symptom burden (*r* =.14–0.53). However, no correlations were observed between SUD, positive affects and FEAR.

### Network analysis

#### Network stability

As highlighted in supplementary Fig. 2 the application of a case-dropping bootstrap technique to evaluate centrality stability yielded a correlation-stability (*CS*) coefficient of 0.75 for the Expected Influence (*EI*) centrality, Strength centrality (*SC*) and Bridge centrality, signifying a high degree of stability. Of note, the bootstrap analysis for edge weights demonstrated that the edges were consistently stable, evidenced by the relatively tight confidence intervals (see supplementary Fig. 3). Hence, the network exhibited high levels of accuracy and stability, making it suitable for dependable analysis.

#### Network estimation

**Fig. 1:**
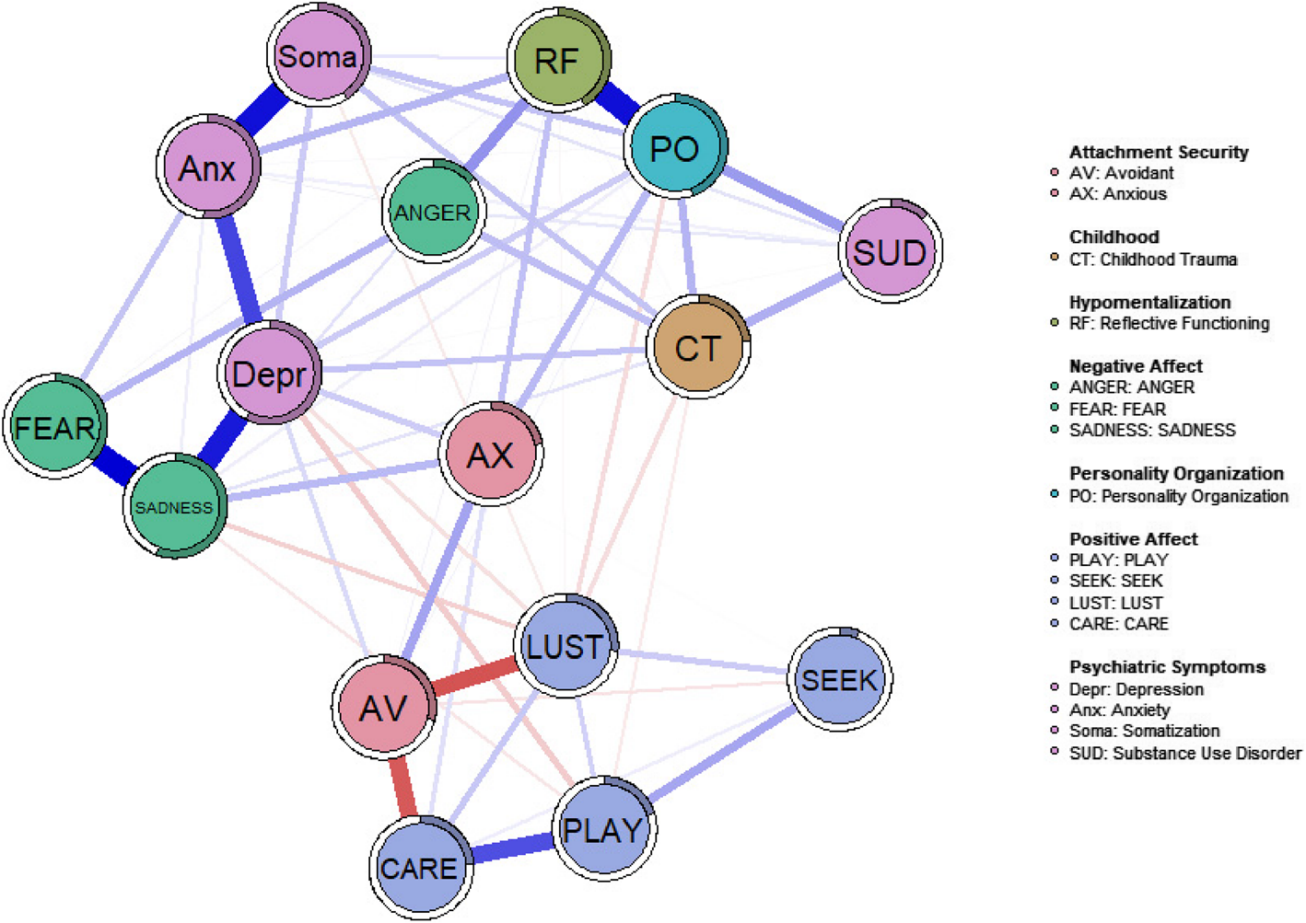
EBICglasso network of childhood trauma, affect, attachment security and psychiatric symptoms visualized via Fruchterman-Reingold algorithm. *Note* Circles around the variables indicate explained variance

Figure 1 visualizes the regularized EBICglasso network plot. As shown childhood trauma showed direct positive relationships with SUD, depression, somatization, ANGER and PO deficits, with PO, childhood trauma and SUD forming a clique. Furthermore, PO, HM and AX formed another clique, with AX connecting PO and HM to AV. In turn, AV was only slightly connected with depression, yet showed strong negative ties to LUST and CARE. As expected, SADNESS was strongly associated with depression and to a lesser degree with AX. Anxiety symptoms showed independent ties to HM and FEAR and strong links to somatization and depression. Both PLAY and LUST were negatively linked to depression. Finally, increased ANGER was connected to deficits in HM and increased FEAR, with the latter linking ANGER to increased SADNESS.

#### Network inference

**Fig. 2:**
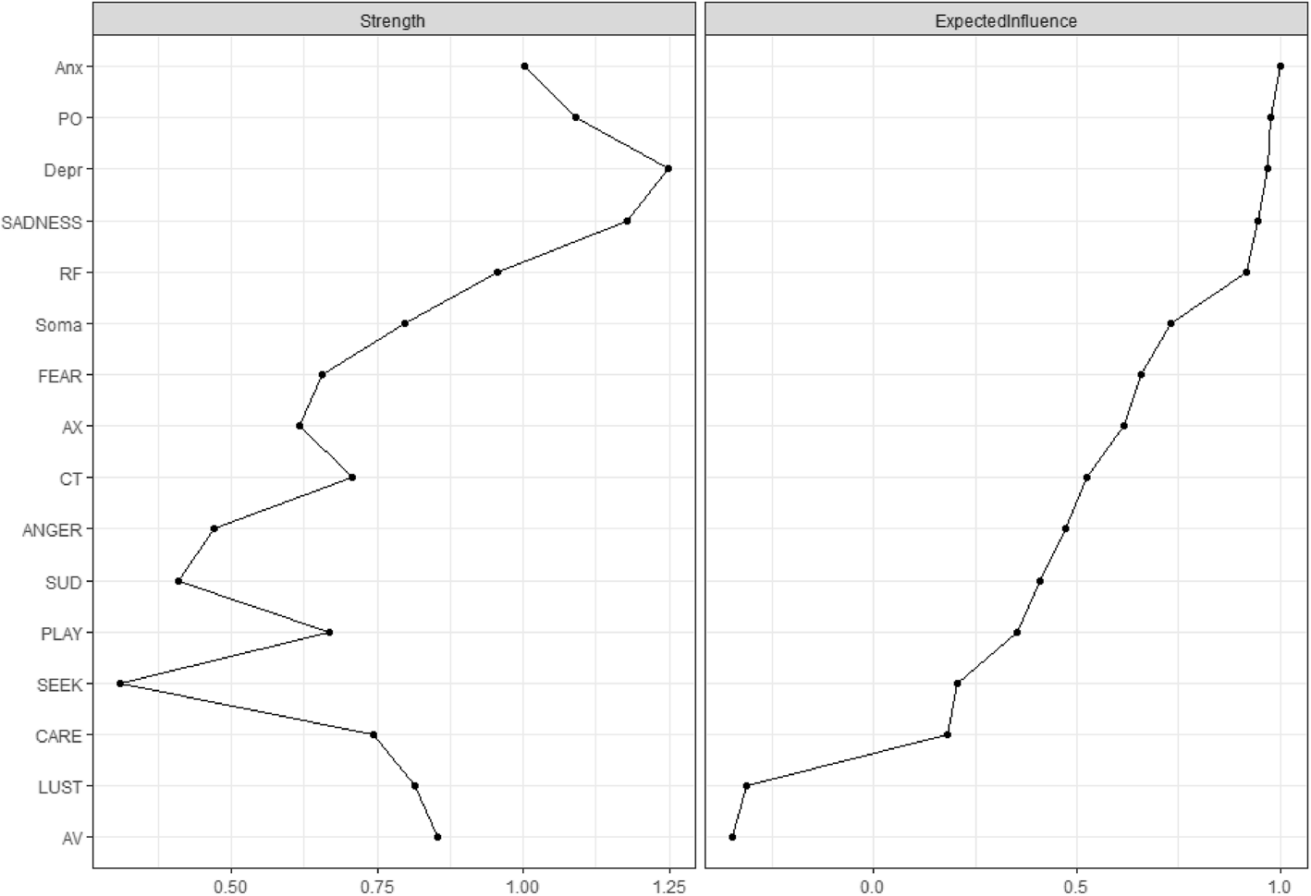
Expected Influence and strength centrality of investigated variables

As shown in Fig. 2 (see supplementary Fig. 4 and supplementary Fig. 5 for the bootstrap difference tests), anxiety (*EI* = 1), PO (*EI* = 0.98), depression (0.97), Sadness (*EI* = 0.95), HM (EI = 0.92) crystallized as most central variables in the investigated network, which did not differ significantly from each other according to bootstrap difference tests (see S5). LUST (*EI* = − 0.31) and AV (*EI* = − 0.35) exhibited an overall inhibiting influence. However, regarding Strength Centrality (*SC*) both LUST (*SC* = 0.81) and AV (*SC* = 0.85) were ranked within the midfield in relation to all other investigated variables. In contrast, ANGER (*EI* = 0.47; *SC* = 0.47), SUD (*EI* = 0.41; *SC* = 0.41) and SEEKING (*EI* = 0.21; *SC* = 0.31) were relatively weakly connected to the rest of the network. In terms of SC, depression (SC = 1.20), SADNESS (*SC* = 1.20), PO (*SC* = 1.10), anxiety (*SC* = 1) and HM (*SC* = 0.95) again were identified as most influential variables.

The average node predictability by all other nodes of the network was *R*^*2*^ = 0.31. The node with the highest predictability was also depression (*R*^*2*^ = 0.58), while SEEKING showed the lowest predictability (*R*^*2*^ = 0.12). The network plot visualizes the predictability scores as pie charts around each node (see Fig. 1).

#### Bridge analysis

**Fig. 3:**
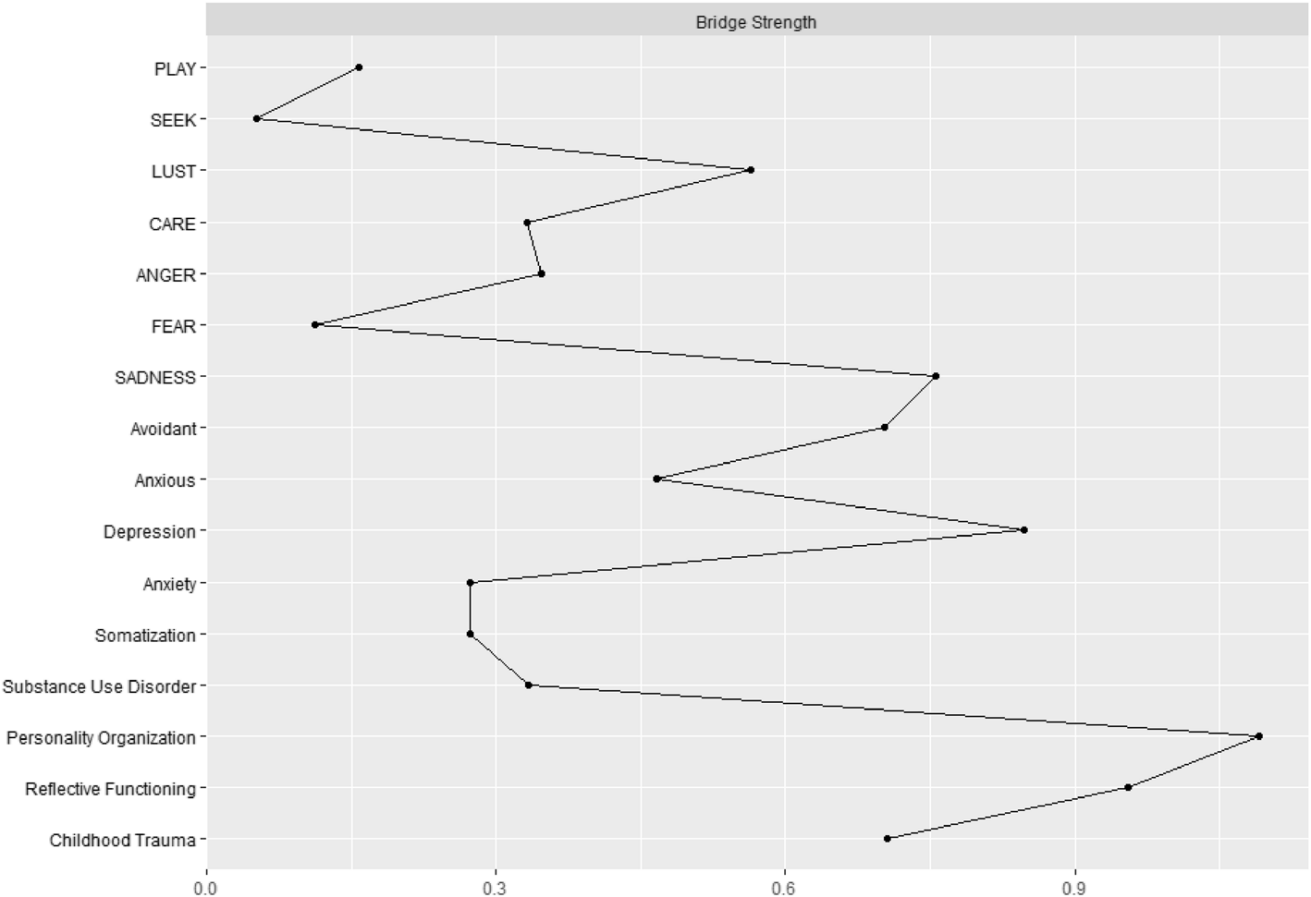
Bridge centrality of investigated variables

Based on the theoretically established communities (see Fig. 1) PO (*BS* = 1.10) and HM (*BS* = 0.95) showed the highest bridge strength within the network (see Fig. 3 for a detailed viszualization). With regard to the bootstrap difference test (see supplementary Fig. 6) PO was a significantly (*p* < 0.05) stronger bridge than depression (*BS* = 0.85), which is in contrast to HM which showed no significant difference to depression. However, HM was a significantly stronger bridge than SADNESS (*BS* = 0.76). SEEKING (*BS* = 0.05), FEAR (*BS* = 0.10) and PLAY (*BS* = 0.16) exhibited the lowest bridge strengths.

#### Relative importance network

**Fig. 4:**
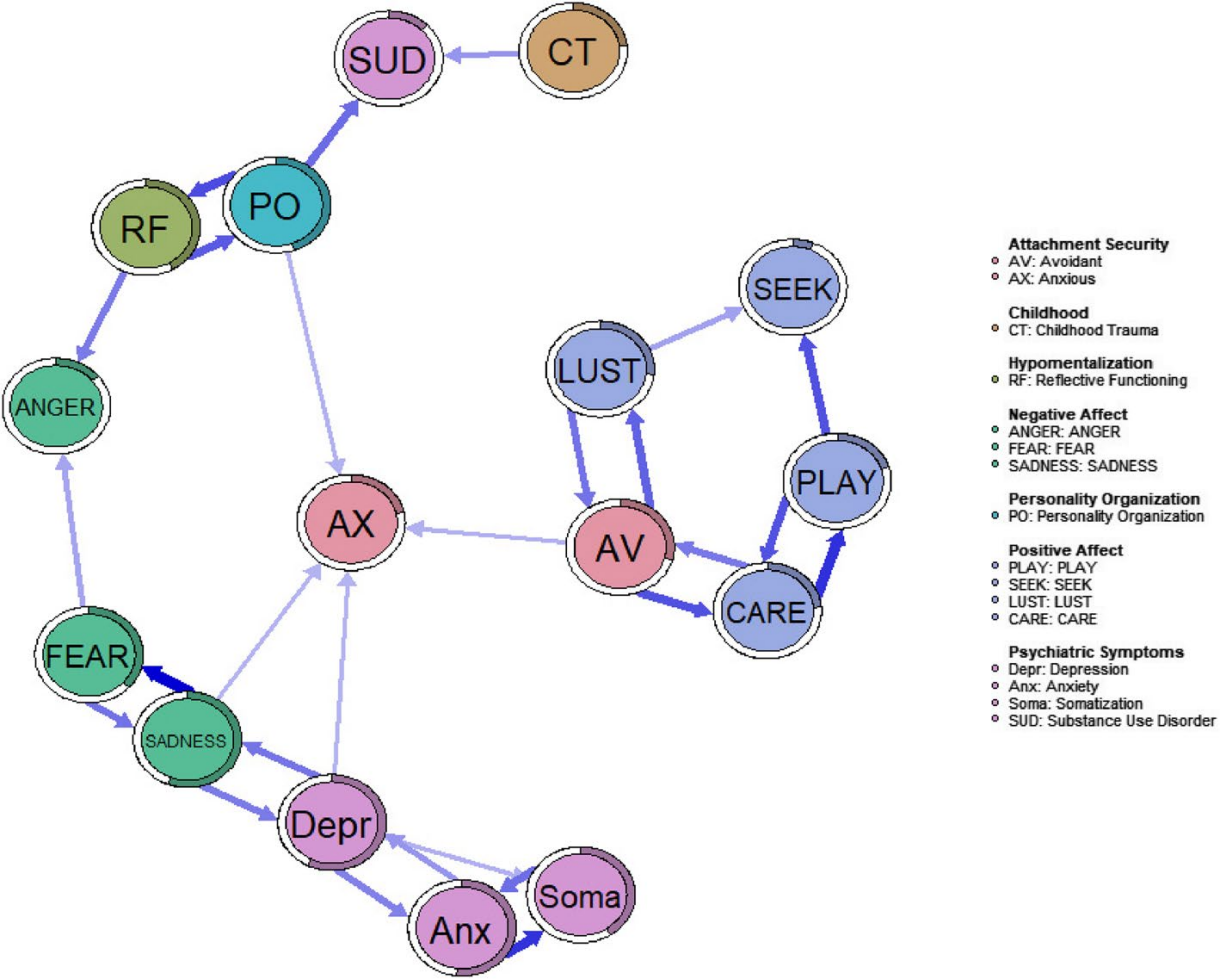
Relative importance network of childhood trauma, affect, attachment security and psychiatric symptoms. Edges below 0.15 are not shown

The relative importance analysis (Fig. 4) suggests that within the investigated network PLAY and CARE, CARE and AV, LUST and AV, PO and HM, depression and SADNESS, SADNESS and FEAR, depression and anxiety and somatization and anxiety are reciprocally associated in a substantial way (*β* > 0.15).

## Discussion

Our study investigated the relationships between childhood trauma, psychodynamic personality concepts (personality organization, reflective functioning, attachment and primary affects) and psychopathological symptoms (depression, anxiety, somatization and substance abuse) within a general population sample. The global results suggest a stable network with personality organization (PO), SADNESS and hypomentalizing (HM) as the most influential nodes in terms of the investigated personality characteristics. This was also reflected in the bridge analysis which estimated PO, HM and SADNESS as most important bridges connecting the a priori assumed conceptual communities (negative affect, positive affect, PO, HM, attachment, psychopathological symptoms and childhood trauma). Finally, results of the relative importance analysis indicated a number of potentially reciprocal connections amongst our studied variables.

Our findings highlight the significant relationship of recalled childhood trauma with various psychodynamic personality constructs and psychopathological symptoms. Specifically, childhood trauma was found to have substantial associations with PO deficits, heightened levels of ANGER, increased substance use disorder (SUD) scores, elevated depressive symptoms and greater somatization. The finding that childhood trauma was linked to deficits in PO, suggests that early adverse experiences are connected to disruptions the development of a cohesive and stable PO. This resonates strongly with object relations theory [29, 32, 101] and previous empirical findings [74, 102].

Regarding the relationship between primary affect traits and childhood trauma, our observations suggest a particularly strong connection between childhood trauma and increased trait ANGER. This finding iterates previous results [103–105], underscoring the detrimental association of perceived childhood maltreatment with affect regulation [106, 107] and its role in developing internalized object relations dominated by aggressive affect [27, 108].

Additionally, our results demonstrated a network of direct links between childhood trauma, PO deficits and higher SUD risk. Previous findings using structural equation modeling estimated that the influence of trauma on SUD is partially mediated via PO [75]. Notably, PO and childhood trauma showed parallel associations with somatization and depression symptoms, highlighting both structural and autobiographical factors in mental health [25, 109].

Aligning with the recent research of Vierl et al. [22] PO was estimated as one of the most central aspects of psychodynamic psychology. The present study expands upon their results by including measures of HM, adult attachment and primary emotion traits. Under the premise that we estimated relatively global dimensions of personality structure and affects, the findings propose PO and HM as particularly promising node for therapeutic outcome research. Whereas self-report psychometric instruments were employed in the present investigation, previous studies utilizing semi-structured interviews to assess personality organization, attachment, and mentalization yielded comparable intercorrelation patterns among these constructs [110, 111].

Moreover, a previous study [74] indicated close links between PO and ANGER. The current results, however, suggest that these ties might be repressed by the stronger link between HM and ANGER. In line with the conceptualization of Fonagy et al. [112], this indicates a crucial role for the ability to think about mental states of oneself and others in affect regulation. The specifically strong ties to the ANGER affect reminds on Bion’s [113] observations on thinking. Here he suggested that the inability to think will lead to an inability to tolerate frustrations, as they are felt as bad or poisoned objects within the body, which the individual seeks to evade. This means the unprocessed affect will be forcefully projected onto and into the objects of the external world, where it can be processed in terms of hostility. Our finding further resonates with the recent study by Fonagy et al. [114], demonstrating the effectiveness of mentalization based therapy in the treatment of convicted patients with antisocial personality disorder.

Regarding the relationship between PO, HM and adult attachment, both deficits in HM and PO were more closely related to attachment anxiety (AX) than to attachment avoidance (AV). Similarly, AX was more closely tied to depressive symptoms and SADNESS, while AV exhibited relatively small associations to psychiatric symptoms. However, AV showed a negative relationship with positive primary affect LUST and CARE. Previous research observed similar patterns in a different general population sample [115]. As the ECR-RD8 measures attachment AV based on (inversely scored) socially desirable items, it is possible that these finding are linked to a methodological artifact. Thus, future studies will have to use the long version of the ECR to clarify this possibility.

In general, the study operated in a conceptual framework inspired by the developments of neuropsychoanalysis, which assumes a mental apparatus which can be roughly categorized into three reciprocally connected layers of primary, secondary and tertiary process systems [58]. While psychometric self-report tools can only estimate the surface texture of these psychoanalytic notions, our approach might still be useful as an approximation towards understanding these nested concepts. The experimental deployment of the *relative importance* algorithm might be able to shed some light on these affiliations: In relation to our total network, established reciprocal relationships were especially prominent between HM and PO. Primary affect SADNESS suggested interdependencies to FEAR and symptoms of depression and attachment AX, highlighting the significance of SADNESS within negative affectivity. The high centrality of SADNESS reaffirms recently published results [115], which presented a very similar finding within a network of psychopathological, affective and attachment related variables. Both observations might be explained by the strong overlap between SADNESS with depressive symptoms and FEAR. Moreover, based on existing literature it is very plausible that high trait SADNESS is significantly interwoven with tendencies towards increased attachment AX [116, 117]. While the current findings underline the centrality of SADNESS, more research will be needed in order to investigate the robustness of this observation in different psychometric networks. Nevertheless, based on the proposed relationship between SADNESS and the endogenous opioid system [118], which in turn has been repeatedly investigated as being of high significance for the human affective system [119–121], the presented results underscore the hypothesis that SADNESS might be a specifically important node in human mental health.

Of note, both LUST and CARE showed a strong mutual relation to attachment AV. Influencing one of the nodes within these dyadic relationships, e.g. in terms of psychotherapy might help to initiate positive cascades regarding improvements in mental health and life satisfaction. However, relative importance network analysis is severely limited if used in the context of cross-sectional data and its result should not be confused with causal relationships but merely reflect relative relations within a given set of variables. Hence, these results should be seen with great caution. Regarding the dissection of the temporal dynamics between these concepts, longitudinal data and the use of corresponding longitudinal network analysis or e.g. (random intercept) cross lagged panel modeling will be necessary (see e.g. 122 for a comparision of different approaches in this regard).

## Limitations and future directions

This study has several limitations that have to be acknowledged. The cross-sectional design restricts the ability to infer causal relationships between the variables, necessitating longitudinal studies to explore the temporal dynamics and establish causality. Additionally, the use of a non-clinical sample may limit the generalizability of the findings to clinical populations, underscoring the need for future research to include diverse clinical samples for validation and broader applicability. Hence, our results should be seen as a proof-of-concept study, which will serve as foundation for further investigations of clinical and longitudinal data. Potential gender differences were not explored, however, multi-group network analysis regarding sex and gender might provide interesting results for future studies. Importantly, the reported centrality measures depend on the investigated network cannot be meaningfully interpreted detached from this context. Moreover, we did not investigate potential empirical communities but assessed the bridge influence based on theoretical considerations. Approaches like exploratory graph analysis (EGA) might complement the understanding regarding empirical clustering [123]. With respect to the ongoing debate regarding the merits of network analysis (see e.g. 124, 125, 126), future research on the relationship between psychodynamic concepts might include the use of both exploratory network analytical techniques (e.g. EGA) and exploratory factor analysis or exploratory structural equation modelling to compare the resulting community and latent factor structures, using confirmatory methods like confirmatory factor analysis.

What is more, results of network analysis have been criticized for their lack of estimation reliability [126]. While we did assess high levels of stability regarding the investigated network, future studies will be needed to insure robustness of these results.

Since its emergence in psychometric research over the last 15 years, psychoanalytic psychology has seen several illustrative examples of the practical applications of network analysis. However, this field is still in its early stages. Further work will help elucidate the often complex conceptual trajectories within depth psychological theory in a straightforward and economical manner, adhering to the principles of empirical research and computational science. Concrete next steps will involve investigating this relationship not only with regard to semi-structured interviews operationalizing PO (e.g. using the STIPO-R; 127), but also investigating this relationship with a higher resolution in order to be able to identify pathways between different subdomains of childhood trauma and PO. The latter could involve dissecting this relationship in terms of subscale interrelations as well as item-level-networks.

Overall, it should be emphasized that the use of brief self-report measures for constructs such as attachment, reflective functioning or personality organization necessarily oversimplifies these complex concepts. Specifically, in contrast to the STIPO, the IPO-16 provides only a rough assessment of personality organization. It primarily captures key features of borderline and psychotic personality organization but does not adequately reflect characteristics of neurotic or normal organization, as conceptualized in Kernberg’s model [128].

Considering our findings highlighting the potential importance of reflective functioning, it may be valuable for future research to explore other contemporary approaches to psychological development and personality pathology, such as epistemic trust, through the lens of network analysis. Specifically, examining the roles of hypomentalizing in relation to epistemic trust regarding political preferences for authoritarian parties and politicians appears particularly promising [129]. This is especially relevant given the close association identified in this study between hypomentalizing and elevated ANGER, which itself is linked to higher trait FEAR.

On the whole, possibilities of analyses using network analytic techniques are very wide (see e.g. 130), spanning from cross-sectional observations in general and clinical population samples to the investigation of longitudinal case studies.

## Conclusions

The findings provide a data-driven, in-depth examination of the complex and often reciprocal relations among psychopathological symptoms, childhood adversity and psychodynamic personality constructs. Our observations highlight critical interconnections among childhood trauma, primary affects, personality functioning and psychopathology. We identified PO, SADNESS and reflective functioning as central nodes within this network, which also exert significant bridge influence. This implies that especially PO, HM and SADNESS might be key nodes for further process-outcome research concerning interventions and therapeutic change.

## Supplementary Information

Supplementary Material 1

## Declarations

### Data availability

The raw data supporting the conclusions of this manuscript will be made available by the authors, without undue reservation, to any qualified researcher.
