# Supplementary material for: Trauma, personality structure and psychopathology: a cartography of psychodynamic constructs
Source: Borderline Personal Disord Emot Dysregul. 2025 Aug 18;12:31. doi: 10.1186/s40479-025-00308-0 (PMC12363119; doi:10.1186/s40479-025-00308-0)
Supplement: Supplementary file 1 — Supplementary Material 1 [file 40479_2025_308_MOESM1_ESM.docx]

| SUPPLEMENTARY TABLE 1: *Descriptive statistics and zero-order correlations for investigated nodes.* | | | | | | | | | | | | | | | | | | |
| --- | --- | --- | --- | --- | --- | --- | --- | --- | --- | --- | --- | --- | --- | --- | --- | --- | --- | --- |
|  |  | **1** | **2** | **3** | **4** | **5** | **6** | **7** | **8** | **9** | **10** | **11** | **12** | **13** | **14** | **15** | **16** |  |
| **1.** | **SEEKING** | - |  |  |  |  |  |  |  |  |  |  |  |  |  |  |  |  |
| **2.** | **LUST** | .22* | - |  |  |  |  |  |  |  |  |  |  |  |  |  |  |  |
| **3.** | **PLAY** | .19* | .33* | - |  |  |  |  |  |  |  |  |  |  |  |  |  |  |
| **4.** | **CARE** | .26* | .30* | .42* | - |  |  |  |  |  |  |  |  |  |  |  |  |  |
| **5.** | **ANGER** | -.06 | -.10 | -.06 | -.07 | - |  |  |  |  |  |  |  |  |  |  |  |  |
| **6.** | **FEAR** | -.06 | -.23* | -.04 | -.16* | .30* | - |  |  |  |  |  |  |  |  |  |  |  |
| **7.** | **SADNESS** | -.13* | -.36* | -.13* | -.27* | .27* | .64* | - |  |  |  |  |  |  |  |  |  |  |
| **8.** | **AV** | -.20* | -.48* | -.43* | -.27* | .15* | .16* | .30* | - |  |  |  |  |  |  |  |  |  |
| **9.** | **AX** | -.02 | -.23* | -.03 | -.09* | .18* | .27* | .41* | .32* | - |  |  |  |  |  |  |  |  |
| **10.** | **RF** | -.12* | -.28* | -.17* | -.15* | .37* | .34* | .43* | .26* | .38* | - |  |  |  |  |  |  |  |
| **11.** | **IPO** | -.12* | -.32* | -.12* | -.14* | .26* | .26* | .44* | .28* | .40* | .63* | - |  |  |  |  |  |  |
| **12.** | **CTQ** | -.08 | -.27* | -.15* | -.21* | .28* | .19* | .37* | .21* | .21* | .34* | .41* | - |  |  |  |  |  |
| **13.** | **Depression** | -.16* | -.38* | -.20* | -.31* | .25* | .44* | .70* | .34* | .41* | .43* | .50* | .42* | - |  |  |  |  |
| **14.** | **Anxiety** | -.06 | -.28* | -.10* | -.18* | .27* | .43* | .53* | .16* | .30* | .47* | .46* | .35* | .66* | - |  |  |  |
| **15.** | **Somatization** | -.08 | -.28* | -.11* | -.19* | .23* | .26* | .37* | .16* | .24* | .42* | .45* | .38* | .53* | .65* | - |  |  |
| **16.** | **WHO-ASSIST** | -.03 | -.11 | -.06 | -.06 | .19* | .09 | .18* | .14* | .15* | .26* | .36* | .31* | .26* | .26* | .27* | - |  |
|  | **Mean** | 3.88 | 3.67 | 3.73 | 3.86 | 2.48 | 3.47 | 2.94 | 1.38 | 12.53 | 18.12 | 29.76 | 41.04 | .94 | .98 | .71 | 18.62 |  |
|  | **SD** | .59 | .78 | .67 | .72 | .77 | .84 | .78 | 5.18 | 5.89 | 7.19 | 8.99 | 14.82 | .88 | .78 | .72 | 25.42 |  |
|  | **Cronbach α** | .69 | .77 | .77 | .65 | .77 | .83 | .81 | .80 | .83 | .81 | .85 | .92 | .85 | .81 | .81 | .93 |  |
| *Notes*. n = 498; * p <.01. | | | | | | | | | | | | | | | | | | |


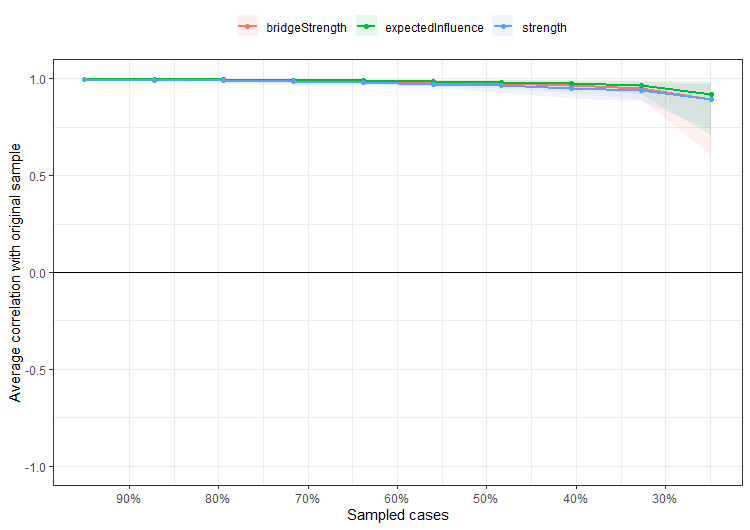


SUPPLEMENTARY FIGURE 1: Case-dropping bootstrap technique to evaluate stability for the Expected Influence centrality, Strength centrality and Bridge centrality.


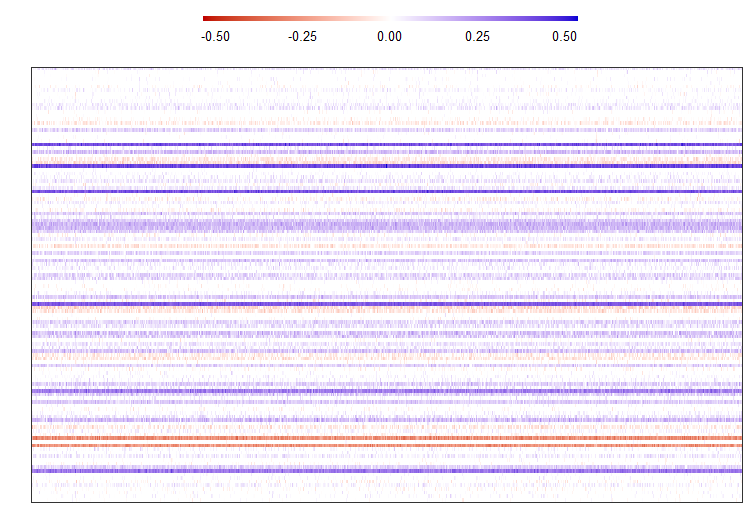


Supplementary Figure 2: In this multiverse plot of bootstrap edge weight results every row indicates an edge and every column a bootstrap. The colour indicates the strength of the edge in each of the 2000 bootstrap replication. The edge weights in our network are stable, because this figure shows fairly straight horizontal lines of the same colour.


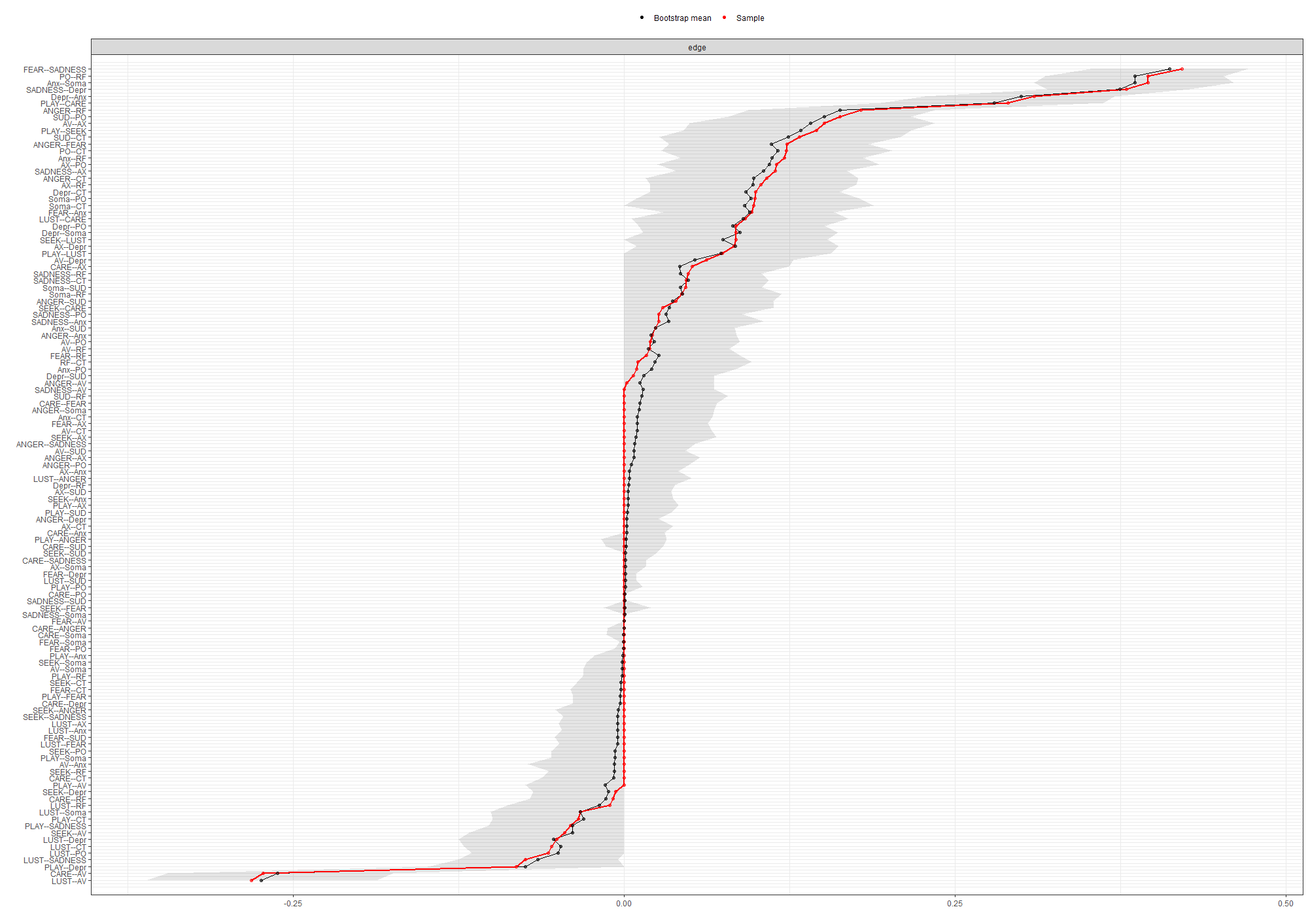


Supplementary Figure 3: Visualization of bootstraped confidence intervals of investigated edge weights in the network across 2000 bootstraps. The red line indicates the original edge weight values, the black line the bootstrap mean edge weight values and the gray-shaded area the bootstrapped 95% CIs of the edge weight values.


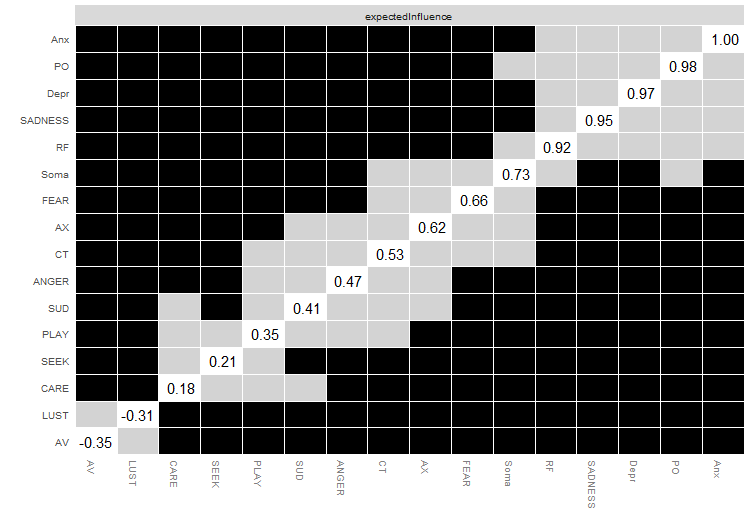


Supplementary Figure 4: The plot shows the differences between all pairs of expected influence. Each row and column represent a node. Black boxes represent significant differences between edge weights (α = .05). Gray boxes indicate non-significant differences.


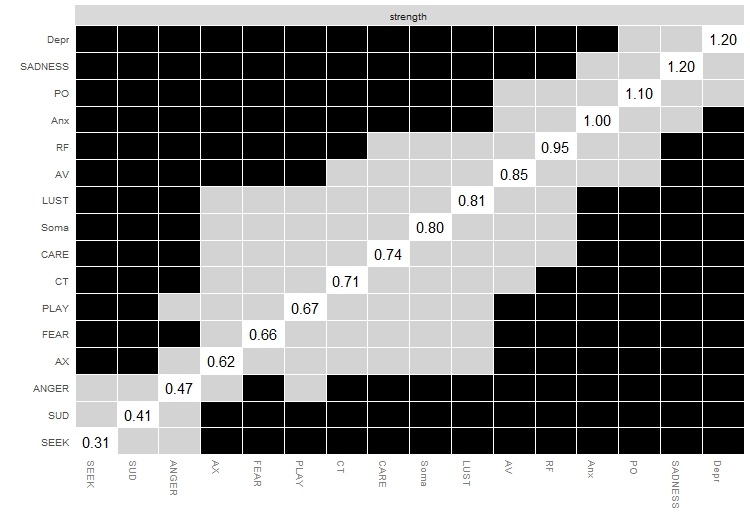


Supplementary Figure 5: The plot shows the differences between all pairs of strength centrality. Each row and column represent a node. Black boxes represent significant differences between edge weights (α = .05). Gray boxes indicate non-significant differences.


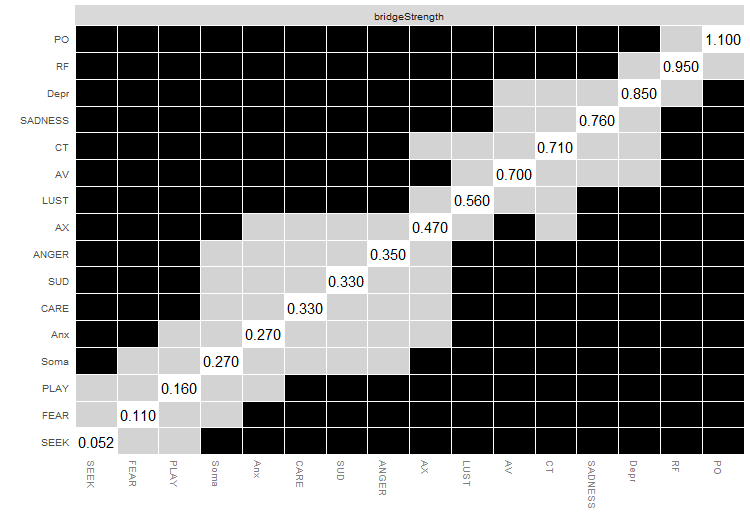


SUPPLEMENTARY FIGURE 6: The plot shows the differences between all pairs of bridge centrality. Each row and column represent a node. Black boxes represent significant differences between edge weights (α = .05). Gray boxes indicate non-significant differences.
